# EUS-guided hybrid rendezvous technique as salvage for standard rendezvous with intra-hepatic bile duct approach

**DOI:** 10.1371/journal.pone.0202445

**Published:** 2018-08-22

**Authors:** Takuji Iwashita, Shinya Uemura, Kensaku Yoshida, Naoki Mita, Ryuichi Tezuka, Ichiro Yasuda, Masahito Shimizu

**Affiliations:** 1 First Department of Internal Medicine, Gifu University Hospital, Gifu, Japan; 2 Third Department of Internal Medicine, University of Toyama, Toyama, Japan; Medizinische Fakultat der RWTH Aachen, GERMANY

## Abstract

EUS-guided rendezvous technique (EUS-RV) is an effective salvage technique for failed biliary cannulation during ERCP. However, it is still difficult to achieve cannulation in some cases, especially using the intrahepatic bile duct (IHBD) approach, which requires complicated guidewire manipulation. EUS-hybrid rendezvous technique (HRV) has been applied as a salvage technique for difficult guidewire placement during EUS-RV with IHBD approach. The aims of this study were to evaluate the efficacy and safety of EUS-HRV using a retrospective study. Database analysis revealed 29 patients who underwent EUS-RV for difficult biliary cannulation. Among them, 8 patients underwent EUS-HRV as a salvage technique for difficult guidewire placement during EUS-RV with the IHBD approach. In EUS-HRV, a 6-French dilator was advanced into the biliary system for better guidewire manipulation. After successful guidewire placement, the EUS scope was exchanged for a duodenoscope, keeping the guidewire and dilator in place. The EUS-placed guidewire was retrieved through the duodenoscope, followed by cannulation over the guidewire. The dilator remained at the fistula until completion of the procedure. The analysis showed that the guidewire placement and the subsequent scope exchange and deep biliary cannulation after the retrieval of the EUS-placed guidewire were successfully conducted for all 8 patients. Mild pancreatitis was recognized as an adverse event in 1 patient. The overall success rate of EUS-RV combined with EUS-HRV was improved up to 90% (26/29). Our results suggested that EUS-HRV can be an effective and safe salvage technique in cases wherein guidewire placement is difficult during EUS-RV with IHBD approach.

## Introduction

Therapeutic endoscopic retrograde cholangiopancreatography (ERCP) for biliary disorders has been widely accepted as a safe, minimally invasive, and efficient procedure. During ERCP, deep biliary cannulation is an inevitable primary step, and its high success rates have been reported. However, an achievement of deep biliary cannulation is occasionally difficult even with the application of advanced cannulation techniques, such as double guidewire or precutting techniques. EUS-guided rendezvous technique (EUS-RV) has also been reported as an effective salvage technique for failed biliary cannulation during ERCP.[1–6] In EUS-RV, the biliary duct is punctured from the intestine under EUS guidance using a needle for fine needle aspiration (FNA), followed by guidewire placement into the duodenum through the needle, biliary duct and ampulla. After guidewire placement, biliary cannulation is re-attempted using the EUS placed guidewire. However, even with the application of EUS-RV, achieving deep biliary cannulation is still difficult in some cases.

**Fig 1:**
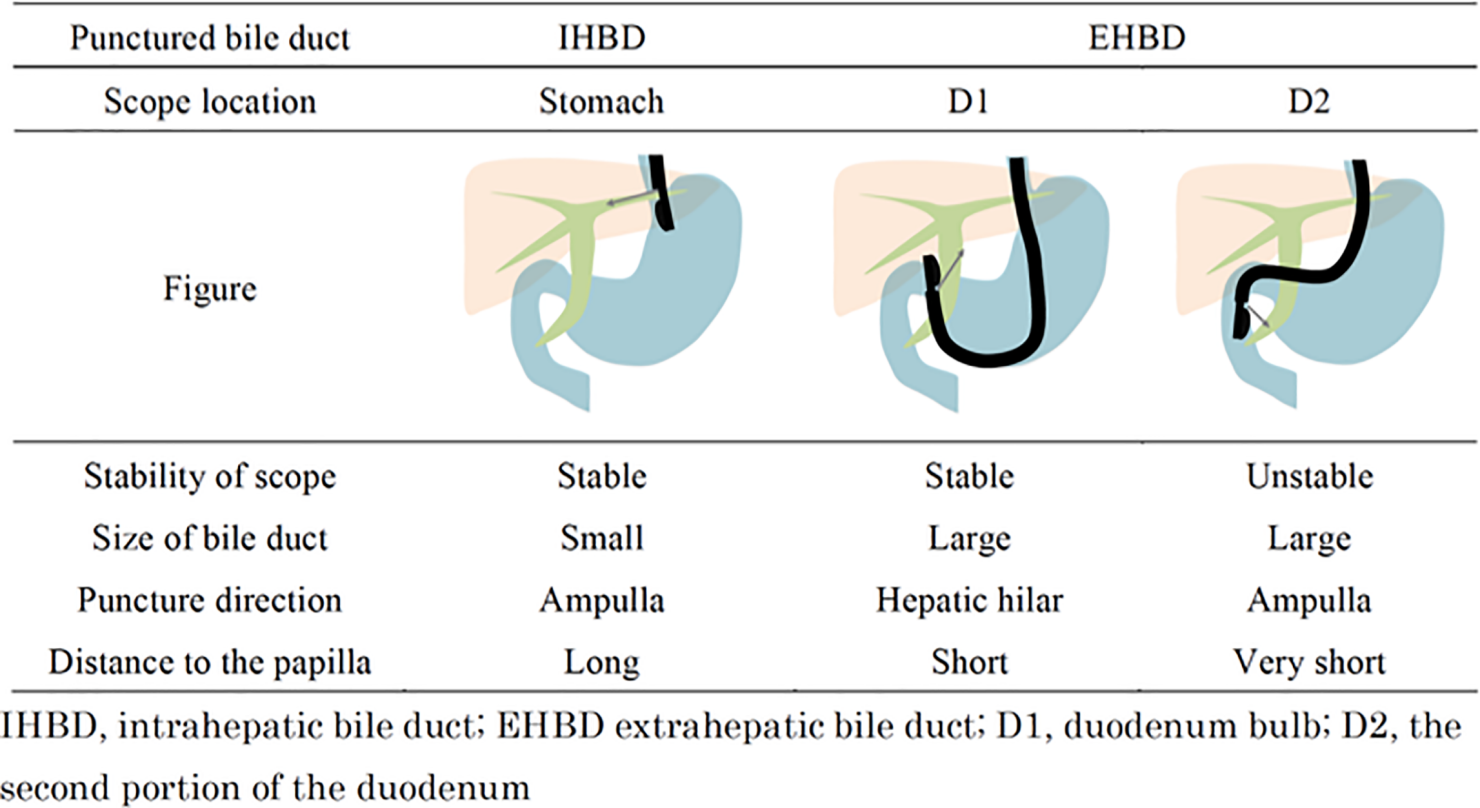
Features of each approach route.

In EUS-RV, the accessed route to the bile duct can be divided into 3 categories based on the biliary ducts accessed and the position of the scope: the intrahepatic bile duct (IHBD) from the stomach, extrahepatic bile duct (EHBD) from the first portion of the duodenum (D1), and EHBD from the second portion of the duodenum (D2) as shown in **Fig 1**.[5] At our institution, EUS-RV using the EHBD from D2 approach has been the first choice to minimize the challenges associated with guidewire manipulation, because this approach has a favorable trajectory for advancing the guidewire toward the distal bile duct and the distance between the biliary access point and the obstruction is minimized.[6] In a previous study, the success rate of EUS-RV with EHBD from D2 approach tended to be higher than those of other approaches, although the EHBD from D2 approach was not always feasible.[6] As for the IHBD from the stomach approach, the longer distance between the needle tip and the ampulla hinders the “pushability” and “torqueability” of the advancing guidewire to overcome downstream resistance.[5, 6] EUS-guided antegrade technique (EUS-AG), which is another EUS-guided technique for bile duct drainage, requires almost the same guidewire burden as the EUS-RV using the IHBD approach. However, in EUS-AG, guidewire placement is relatively straightforward, since the guidewire can be manipulated by the coordinated movement of a catheter inside the biliary system, similar to the percutaneous transhepatic biliary (PTB) approach.[7–12]

A novel EUS-hybrid rendezvous technique (EUS-HRV), wherein a dilator was temporally inserted into the biliary system to maintain guidewire maneuverability like EUS-AG, has been applied as a salvage technique in cases where the guidewire placement was considered difficult or impossible during the EUS-RV with IHBD approach. We therefore conducted this retrospective study to evaluate the efficacy and safety of EUS-HRV.

## Patients and methods

### Patient’s selection

This was a retrospective study conducted in a single academic center, Gifu University Hospital. Retrospective database analysis of all ERCP and EUS-guided procedures was performed between April 2012 and March 2017. We included patients who underwent EUS-RV or HRV for failed biliary cannulation, but excluded patients with the following conditions: surgically altered upper intestinal anatomy (except for Billroth I) or management with combination of percutaneous or surgical approach. During the study period, 1398 patients with normal upper intestinal anatomy underwent ERCP for biliary diseases. Among them, achievement of deep biliary cannulation was failed in 33 patients. Four patients who were managed by percutaneous approach were excluded from the analysis. Finally, 29 patients underwent EUS-RV or EUS-HRV as a salvage technique for difficult biliary cannulation were included and analyzed in this study. No patients were managed by a surgical approach in the study period. Written informed consent for ERCP and EUS-guided procedures was obtained from all patients.

### EUS-guided hybrid rendezvous technique

**Fig 2:**
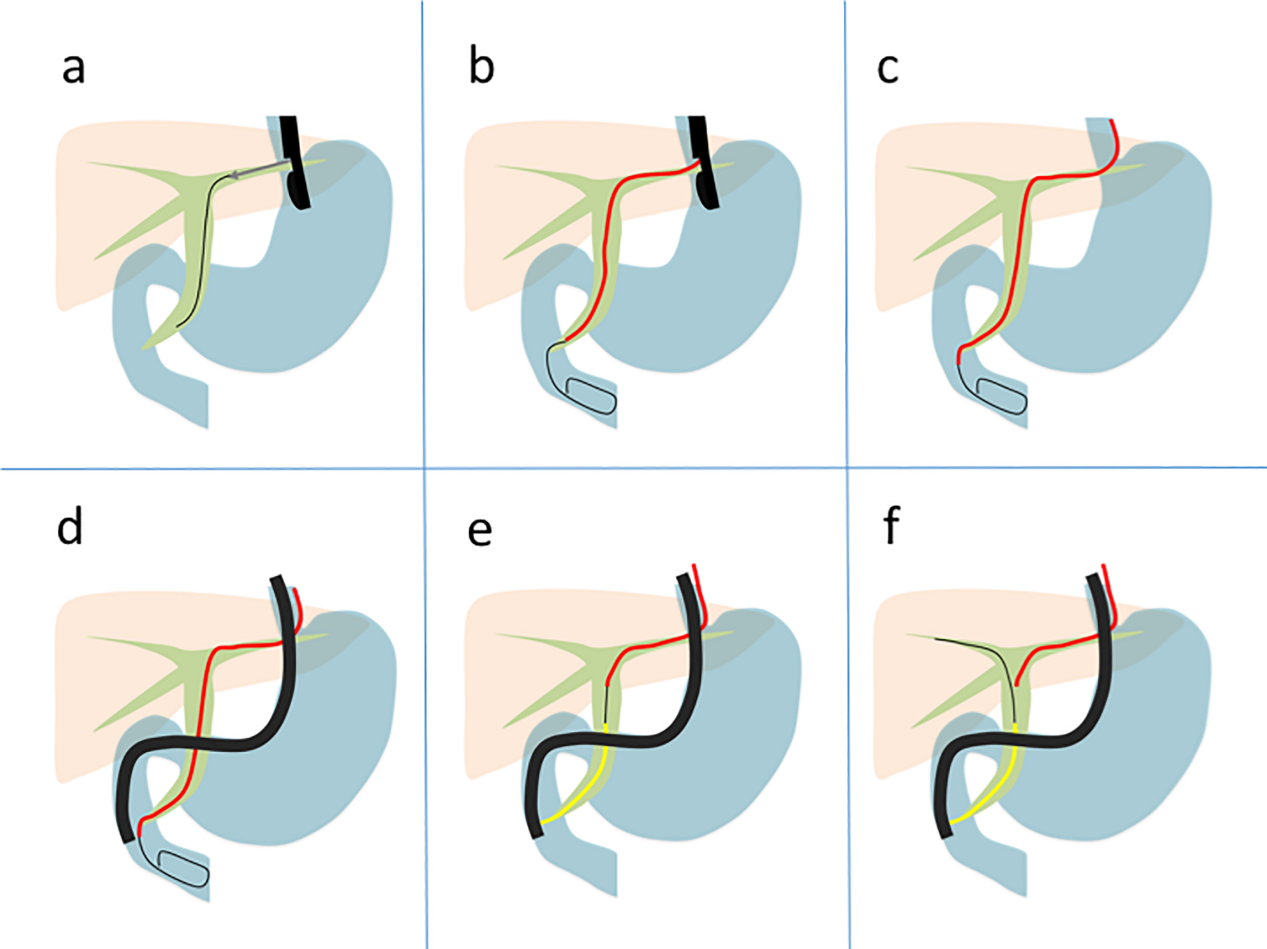
Actual technique of hybrid rendezvous technique. **a**, The intrahepatic bile duct was punctured under EUS guidance, followed by guidewire placement into the bile duct through the needle. **b**, Dilation of the fistula using a 6-French bougie dilator (red line), followed by guidewire manipulation into the duodenum through the ampulla with improved pushability and torqueability of the guidewire., **c**, The EUS scope was removed, keeping the guidewire and dilator in place. **d**, A duodenal scope was inserted into the duodenum and the EUS-placed guidewire was retrieved through the scope with a snare or loop cutter. During the retrieval, the guidewire could be manipulated through the dilator to assist the retrieval. **e**, A deep biliary cannulation (yellow line) was achieved over the EUS-placed guidewire. **f**, The dilator remained at the fistula until the completion of the originally planned therapeutic procedure.

**Fig 3:**
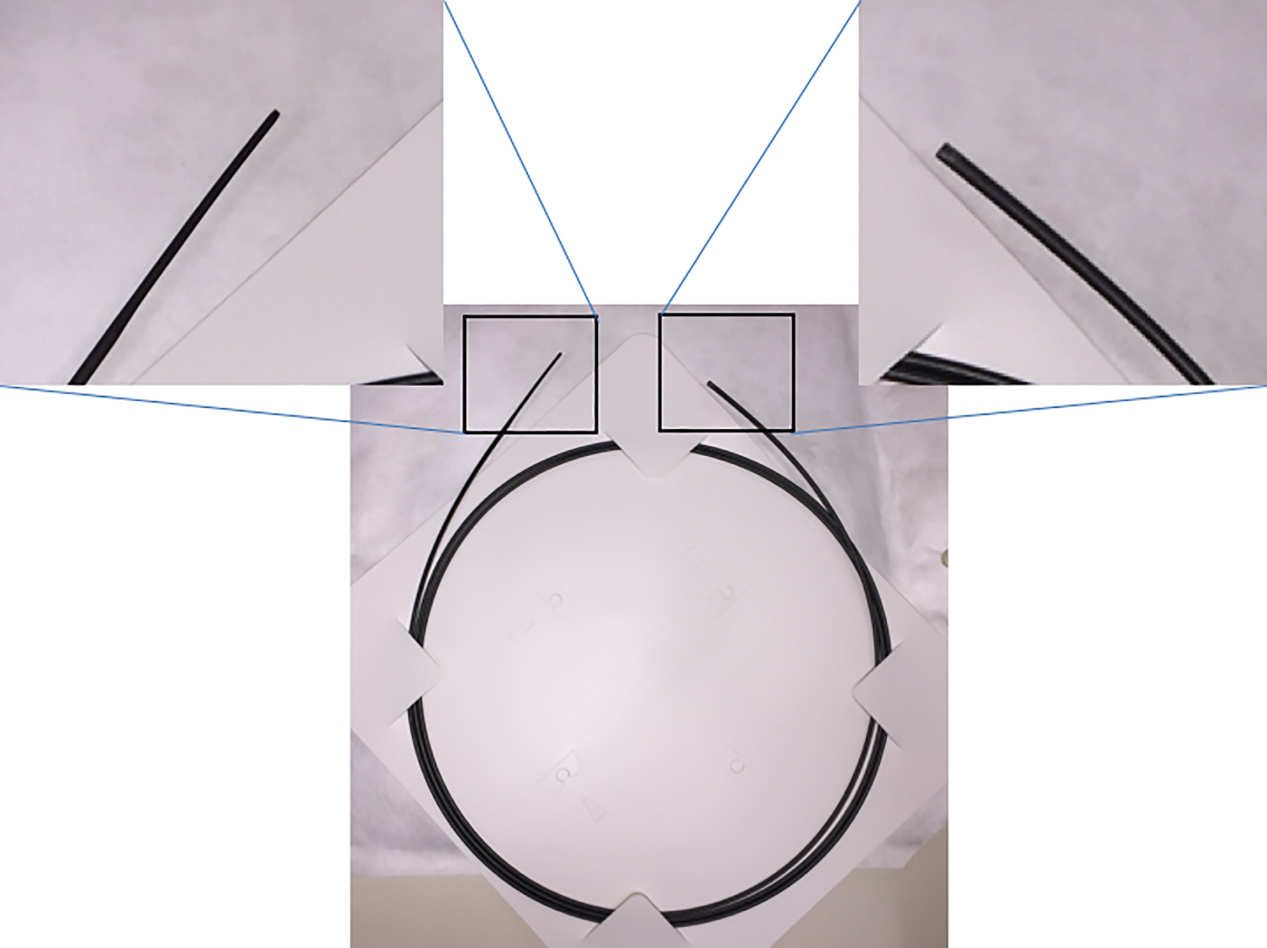
A 6-French dilator with well-tapered tip but without any fixed attachment at the end.

ERCP was performed under moderate sedation and prophylactic broad-spectrum antibiotics were administered prior to the procedure in all patients. All EUS-guided interventions were performed by 2 experienced endoscopists (TI and IY) during the study period. EUS-RV was attempted after failed deep biliary cannulation. Once the decision to perform EUS-RV was made, the ERCP scope (TJF-260V; Olympus Co., Tokyo, Japan) was exchanged with the EUS scope (UCT-260; Olympus Co.). The biliary system was evaluated from the stomach, D1, and D2 with EUS before the puncture. At our institution, the EHBD from D2 approach was chosen as the primary approach if it was technically and anatomically possible, followed by the IHBD from the stomach and the EHBD from D1 approaches. The bile duct was subsequently punctured using a 19-gauge FNA needle (SonoTip Pro Control; Medi-Globe GmbH, Achenmuhle, Germany) primed with a contrast agent, and proper puncture was confirmed with a cholangiogram through the needle. A 0.025 guidewire (VisiGlide or VisiGlide 2; Olympus Co.) was inserted into the biliary system through the needle and manipulated into the duodenum via the ampulla (**Fig 2A**). EUS-HRV was utilized if the guidewire placement into the duodenum was difficult or impossible because of downstream resistance during the IHBD approach from the stomach. A 6-French bougie dilator (PD-SS6F180C; Gadelius Medical, Tokyo, Japan; **Fig 3**) with well-tapered tip for smooth dilation but without any attachment at the end, such as a nasobiliary drainage tube, was inserted into the biliary system during the EUS-HRV procedure (**Fig 2B**). The guidewire was manipulated again with improved torqueability and pushability due to the support from the dilator, similar to the PTB approach or EUS-AG. Once the guidewire was inserted into the duodenum, the EUS scope was retrieved, keeping both the guidewire and the dilator in place (**Fig 2C**). The ERCP scope was inserted into the duodenum next to the dilator. (**Fig 2D**) After identification of the dilator and the guidewire coming out from the ampulla in the duodenum, the guidewire was grasped by a snare or loop cutter. At this point, the EUS placed guidewire could be freely manipulated through the dilator, since the dilator worked like a catheter. The guidewire was retrieved through the accessory channel of the duodenoscope. Deep biliary cannulation was performed using the ERCP catheter over the EUS-placed guidewire (**Fig 2E**). After deep biliary cannulation, the EUS-placed guidewire was removed through the dilator, and another guidewire was inserted into the biliary system through the ERCP catheter. At this stage, the dilator was pulled back slightly within the biliary system but was not removed until the completion of the whole procedure to reduce possible bile leak through the fistula (**Fig 2F**). Subsequently, the originally-planned endoscopic therapy was performed (S1 **Video**).

### Outcomes

The primary outcome of this study was the technical success rate of EUS-HRV as a salvage technique to achieve deep biliary cannulation during ERCP. The secondary outcome was the adverse event rate and the overall success rate of EUS-RV combined with EUS-HRV. The technical success was defined as an achievement of deep biliary cannulation during ERCP. Early adverse events were also evaluated according to a lexicon for endoscopic adverse events of the American Society of Gastroenterological Endoscopy.[13] The procedure time for EUS-HRV was measured from the time of insertion of the EUS scope to the time of successful or failed cannulation of EUS-HRV. Continuous variables were presented as median and range. Statistical analyses were performed using the Mann–Whitney U test for continuous variables and the Fisher exact test for categorical variables. A 2-sided P value <0.05 was considered statistically significant. All statistical analyses were performed using JMP version 13.0.0 (SAS Institute, Cary, NC). This study protocol was approved by the Institutional Review Board of Gifu University Hospital on October 4th, 2017 (29–229). This study was registered at UMIN Clinical Trials Registry (UMIN000030544). The consent of participation of patients in this study was obtained through an opt-out methodology. This study was conducted in accordance with the Declaration of Helsinki.

## Results

Among 29 patients who underwent EUS-RV for failed biliary cannulation after the median number of 15 attempts (range 4–35) of cannulation with the median time of 15 minutes (range 5–47) during the study period, 13 patients underwent EUS-RV with IHBD approach. EUS-HRV was attempted in 8 patients (3 males, median age, 70.5 years [range: 51–91 years]) because of the difficulty in guidewire placement during EUS-RV with IHBD approach from the stomach. ERCP was performed to manage malignant biliary obstruction in 7 patients and common bile stone in 1 patient. The reasons for difficult biliary cannulation were cancer invasion in 4 patients, periampullary diverticulum in 1 patient, and other technical difficulties in 3 patients. During EUS-RV, the median diameter of punctured IHBD was 4 mm with a range of 3–5 mm (**Table 1**).

**Table 1:** Details of patients and procedures.

	Age (yo)	Sex	Biliary diseases	Reason for failed biliary cannulation	Size of punctured bile duct (mm)	Successful dilator insertion	Success of HRV	Treatments	Required time for HRV	Adverse event
1	82	Female	MBO	technical	4	Yes	Yes	Naso-billiary drainage	47	Mild pancreatitis
2	68	Male	MBO	duodenal invasion	3	Yes	Yes	Metallic stent	40	No
3	91	Female	MBO	technical	4	Yes	Yes	Metallic stent	35	No
4	67	Female	MBO	duodenal invasion	5	Yes	Yes	Plastic stent	17	No
5	73	Male	MBO	duodenal invasion	4	Yes	Yes	Plastic stent	44	No
6	89	Male	CBDS	diverticulum	3	Yes	Yes	EPLBD and stone removal	25	No
7	51	Female	MBO	duodenal invasion	5	Yes	Yes	Metallic stent	30	No
8	65	Female	MBO	technical	3	Yes	Yes	Plastic stent	35	No

MBO, malignant biliary obstruction; CBDS, common bile duct stone; EPLBD, endoscopic papillary large balloon dilation; HRV, hybrid-rendezvous technique

**Fig 4:**
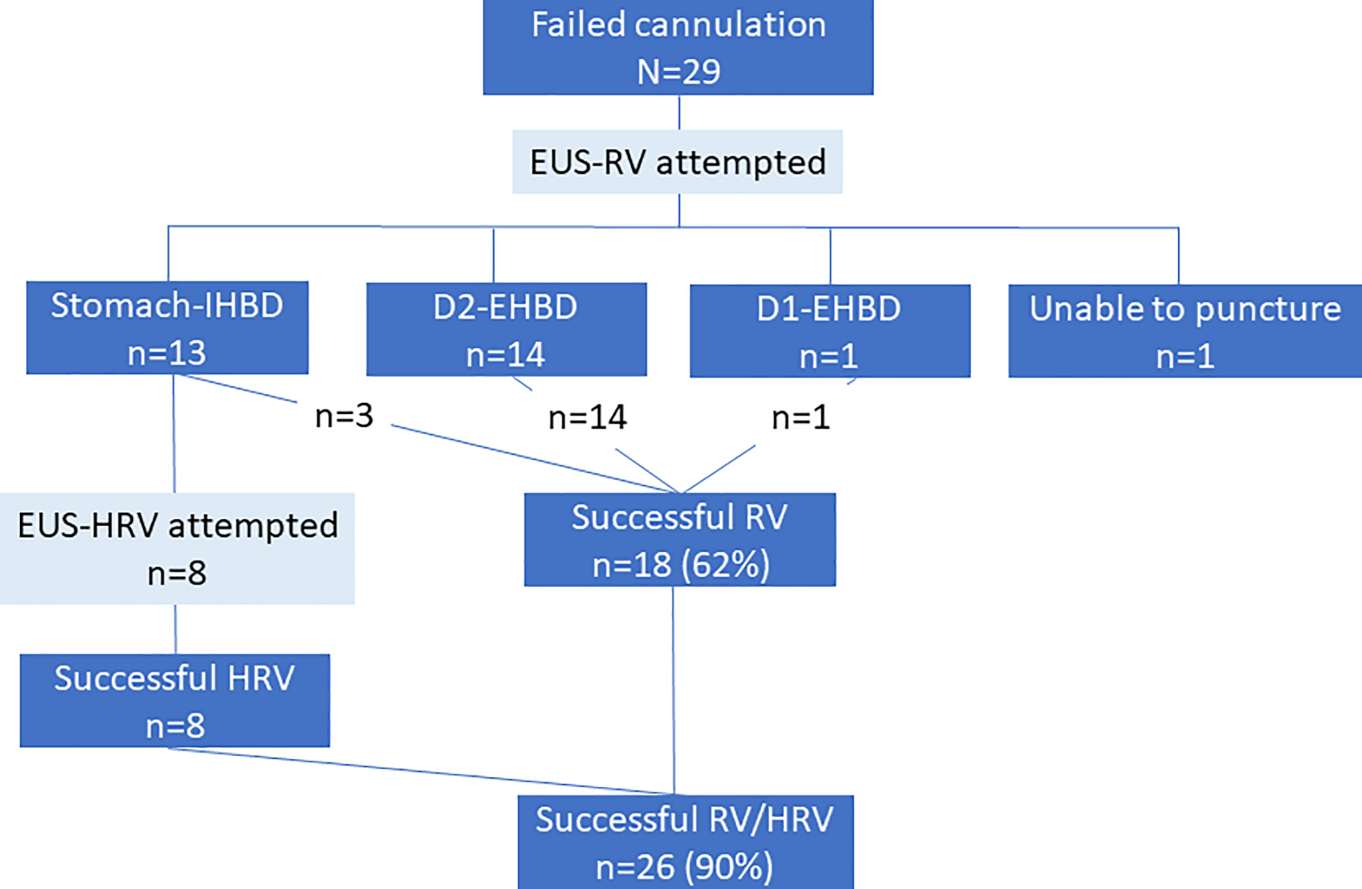
Flow of the patients in endoscopic ultrasound-guided rendezvous and hybrid rendezvous techniques.

Dilator insertion into the duodenum and guidewire manipulation was successfully performed in all patients who underwent EUS-HRV. The subsequent scope exchange and deep biliary cannulation after retrieval of the EUS-placed guidewire was also successfully conducted in all 8 patients. After deep biliary cannulation was achieved, bile disorders were managed through metallic stent placement in 3 patients, plastic stent placement in 3 patients, nasobiliary drainage tube placement in 1 patient, and endoscopic papillary large balloon dilation with stone removal in 1 patient. The median procedure time for EUS-HRV was 35 minutes (17–47 minutes). Mild pancreatitis was recognized in 1 patient and was successfully managed conservatively; however, no other adverse events were observed in the remaining 7 patients (**Table 1**). The procedure time and adverse event rates in EUS-RV were 32.5 minutes (14–89) and 14% (3/21: temporal abdominal pain, liver hematoma, and guidewire fracture) and did not show significant differences in comparison with EUS-HRV (p = 0.78 and p = 1.00), respectively. The overall success rate of EUS-RV combined with EUS-HRV was improved up to 90% (26/29) (**Fig 4**).

## Discussion

In this retrospective study, EUS-HRV was attempted in 8 patients as a salvage technique for difficult or impossible guidewire placement during EUS-RV with IHBD approach and was successful in achieving deep biliary cannulation in all patients (100%). The adverse event rate was 12.5% (1/8), which was similar to the overall adverse event rate in the previous pooled analysis (11%, 24/217). EUS-RV combined with EUS-HRV improved the success rate of biliary cannulation up to 90%.

In EUS-RV, there are 3 technically challenging steps: biliary puncture, guidewire placement, and biliary cannulation using the EUS placed guidewire.[4] Guidewire placement is considered to be the most challenging step, because the guidewire has to be manipulated through the FNA needle into the intestine via the biliary system and ampulla. In addition, there are technical limitations in guidewire maneuverability, due to the long rigid FNA needle and the limited angulation of the needle itself within the bile duct.[4] EUS-AG also requires almost the same guidewire burden same as EUS-RV; however, the guidewire manipulation is not as challenging as that of EUS-RV, since an ERCP catheter can be inserted into the biliary system to keep better pushability and torqueability of the guidewire. This additionally allows the endoscopists to obtain a cholangiogram.[12] Taking into account these features of each technique, we combined EUS-RV with EUS-AG as HRV in terms of guidewire manipulation in conjunction with the dilator inside of biliary system. In this study, EUS-HRV was actually applied in 8 patients with failed guidewire placement during EUS-RV with IHBD approach due to a longer distance between the puncture point and downstream resistances. HRV could successfully salvage all patients with improved pushability and torqueability of guidewire by the inserted dilator.

We noticed an additional advantage of EUS-HRV in obtaining biliary cannulation using the EUS-placed guidewire. In this step, there are 2 methods to obtain biliary cannulation, the “along the wire” or “over the wire” methods.[14] In the “along the wire” method, deep biliary cannulation is attempted along the EUS-placed guidewire, similar to a regular biliary cannulation. In the “over the wire” method, the EUS-placed guidewire is retrieved through the accessory channel of the ERCP scope with a snare or loop cutter followed by biliary cannulation over the retrieved guidewire. In the former method, retrieval of the guidewire is omissible, but biliary cannulation is more challenging since cannulation is attempted just along the EUS-placed guidewire. In the second method, cannulation is straightforward once the guidewire is retrieved through the endoscopic accessory channel, although the retrieval itself is time-consuming and there is a risk of losing the guidewire, since the EUS-placed guidewire has to be pulled back for grasping the tip of the guidewire. However, in EUS-HRV, the EUS-placed guidewire can be freely manipulated during the retrieval of the guidewire through the channel of the ERCP scope, because the remained dilator within the biliary system through the fistula enables manipulation of the guidewire from the outside of the body.

The possible disadvantage of EUS-HRV is that the fistula dilation using a bougie dilator might increase the risk of bile leakage into the abdominal cavity. However, we believe this risk is minimal because the dilator is maintained at the site of the fistula until completion of the originally planned procedures, such as stone removal or stent placement. This can potentially seal the fistula, thus preventing bile leakage into the abdominal cavity. After the procedure, the dilated fistula should close shortly and spontaneously, as the internal pressure of the bile duct should be well controlled by the proper endoscopic management of the underlying biliary diseases and tamponade effect of liver parenchyma. No bile peritonitis was recognized in this study. Presently, however, we are unsure whether EUS-HRV can be safely applied for EUS-RV with EHBD approach route that do not have liver parenchyma at the puncture site. Another possible disadvantage of EUS-HRV is the increased risk of bleeding due to the dilation of the fistula, although we also believe this risk is minimal because the fistula is dilated using a 6-French mechanical bougie dilator without any diathermic or balloon dilation.

PTB-RV is another option to salvage failed biliary deep cannulation during ERCP. PTB-RV has several potential advantages and disadvantage over EUS-RV. PTB-RV can reduce the distance between the operator’s hand and the ampulla and allows the use of flexible percutaneous guiding devices, which enhances support for guidewire manipulation. [15] Furthermore, PTB-related techniques might be more widely available than EUS-related techniques, given the conventionality of PTB-techniques. Potential disadvantages of PTB-RV include: PTB-approaches traditionally require temporal external drainage tube placement with a multi-step approach that can cause patient discomfort and tube-related adverse events. Advantages of EUS–RV over PTB-RV include: EUS–RV can be performed in the same setting and single session as the ERCP procedure without changing the patient’s posture. Furthermore, by the application of EUS-HRV, this technique can improve guidewire maneuverability by insertion of a dilator into the biliary system, which is similar to the PTB-approach.

This study has several limitations. Patient selection might have been biased because of the retrospective nature of the study design. Internal and external validity also might be low, as this was a single-center study with a small sample size.

In conclusion, EUS-HRV can be an effective and safe salvage technique in cases where guidewire placement is considered difficult during EUS-RV with IHBD approach to improve the success rate of EUS-RV. Further prospective studies involving a larger cohort are warranted to confirm the findings of this study.

## Supporting information

S1 Video Endoscopic ultrasound-guided hybrid rendezvous technique. (3GP)

## Abbreviations

ERCP: endoscopic retrograde cholangiopancreatography
EUS-RV: endoscopic ultrasound-guided rendezvous technique
FNA: fine needle aspiration
IHBD: intrahepatic bile duct
EHBD: extrahepatic bile duct
D1: the first portion of the duodenum
D2: the second portion of the duodenum
EUS-AG: endoscopic ultrasound-guided antegrade technique
EUS-HRV: endoscopic ultrasound-guided hybrid rendezvous technique
PTB: percutaneous transhepatic biliary
